# Arming Anti-EGFRvIII CAR-T With TGFβ Trap Improves Antitumor Efficacy in Glioma Mouse Models

**DOI:** 10.3389/fonc.2020.01117

**Published:** 2020-08-18

**Authors:** You Li, Huifang Wu, Gang Chen, Xiaofan Wei, Chunyu Wang, Shanshan Zhou, Ailing Huang, Zui Zhang, Changyou Zhan, Yanling Wu, Tianlei Ying

**Affiliations:** ^1^MOE/NHC/CAMS Key Laboratory of Medical Molecular Virology, School of Basic Medical Sciences, Shanghai Medical College, Fudan University, Shanghai, China; ^2^School of Life Science and Technology, Tongji University, Shanghai, China; ^3^State Key Laboratory of Molecular Engineering of Polymers, Department of Pharmacology, School of Basic Medical Sciences, Fudan University, Shanghai, China

**Keywords:** glioblastoma, TGFβ, CAR-T, EGFRvIII, TGFRII

## Abstract

Glioblastoma (GBM) is an aggressive malignancy with poor prognosis. New therapeutic strategies for GBM are urgently needed. Although clinical studies have demonstrated the feasibility and safety of chimeric antigen receptor (CAR) T cell therapy for GBM, its efficacy has not been that impressive. The major limitation for anti-tumor efficacy of CAR-Ts is the immunosuppressive milieu of the GBM tumor microenvironment (TME). TGFβ, a substantial component in GBM, compromises the immune response and contributes to immune evasion and tumor progression. To overcome this limitation and improve the efficacy of CAR-T cells for GBM, we optimized an EGFRvIII-specific CAR construct with TGFRII ectodomain as a TGFβ-trap and generated TGFβ-resistant CAR-Ts for GBM therapy. We demonstrated that this TGFβ-trapped architecture enhanced anti-tumor efficacy of EGFRvIII-specific CAR-T and prolonged the survival of mice bearing GBM. In addition, the GBM-infiltrated microglia, typically considered tumorigenic, showed increased expression of M1 polarization markers after treatment with the TGFβ-trap CAR-Ts group, indicating that these microglia were polarized toward a pro-inflammatory and anti-tumorigenic phenotype. Overall, these results indicated that arming CAR-T cells with a TGFβ-trap diminishes the immunosuppressive effect and is a potential strategy to improve CAR-T efficacy for GBM therapy.

## Introduction

Glioblastomas (GBM, grade IV) are by far the most common and malignant type of tumors that develop in the central nervous system (1). Despite the standard treatment of total surgical resection followed by radiotherapy and adjuvant chemotherapy, GBM almost invariably recurs and the 5-years overall survival (OS) rate remains <10% (2, 3). Although Bevacizumab was approved by FDA for use in recurrent GBM in 2009, the clinical responses of anti-VEGF treatment were demonstrated to be transient with no improvement in OS (4). Thus, alternative therapeutic strategies for GBM are urgently needed. The successes of immunotherapy in other cancers has increasingly generated interest in it as an attractive therapeutic strategy for treatment of glioblastomas. The first large phase III trial of PD-1 blockade Nivolumab for GBM (CheckMate 143) was initiated in 2014. However, it did not meet its primary endpoint of significant improvement of overall survival in GBM patients.

Chimeric antigen receptor (CAR) T cells therapy provides an alternative promising approach to checkpoint blockades. CAR-T cells are *ex vivo* generated, which is independent of endogenous responses and pre-existing anti-tumor immunity, and thus bypasses the defective immune system. Clinical successes in hematological malignancies (5, 6) demonstrated the power of CAR-T therapy and extended the potential to develop CAR-T therapy targeting GBM. Several clinical trials with CAR-T therapy targeting Her-2, IL13R, or EGFRvIII are ongoing for GBM to reduce recurrence rates (6–8). Nevertheless, given the complex tumor microenvironment (TME) of glioma characterized by immune exclusion, or suppressive state and various immune escape mechanisms (9), immunotherapies including CAR-T therapy on GBM has shown equivocal progress so far (10).

To improve the therapeutic efficiency in GBM, circumventing the immunosuppressive TME is especially imperative for CAR-T therapy. One approach is to optimize CAR constructs, simultaneously targeting the TME with innovative engineering strategies to diminish the inhibition on CAR-T. Among the multiple pathways and milieu that compromise the immune response in the tumor microenvironment, TGFβ, as a potent immunosuppressant cytokine, plays a crucial role (11, 12). It has been reported that TGFβ expression is dramatically increased in histological GBM tissues of patients and the elevated TGFβ is strikingly correlated with a high tumor grade (13, 14). Indeed, TGFβ expression in malignant brain tumors excludes T cell infiltration, suppresses the anti-tumor immune response and promotes tumor survival (15). Numerous pre-clinical studies have shown that blocking TGFβ signaling could reverse the suppressive immunologic environment and benefit therapeutic efficacy in GBM by blocking TGFβ signaling (16–18). Thus, our strategy is to combine CAR-T therapy with TGFβ targeting as the next generation of CAR-T for GBM therapy.

Epidermal growth factor receptor variant III (EGFRvIII), characterized by deletion of exons 2 through 7 with insertion of a glycine residue at the junction between exon 1 and 8 of EGFR, is a consistent tumor specific mutation that is widely expressed in GBM, with an overall prevalence of 20–30% in GBM patients (19, 20). In addition, the presence of EGFRvIII-expressing cells confers a negative prognosis for patients with GBM (21, 22). Therefore, this EGFRvIII mutation was established as an eligible target for the development of humoral- or cell-mediated immunotherapy in GBM.

TGFβ receptor ectodomain (TGFRII ECD) can function as a TGFβ trap with high affinity to TGFβ1, TGFβ2, and TGFβ3. M7824, the PD-L1/TGFβ targeting fusion protein from Merck, has demonstrated that a TGFβ trap can promote the expression of immune cell signature and improve anti-tumor immunity (23). This suggests that TGFRII ECD could be engineered as a TGFβ-trap onto CAR-T cells to reduce the immune suppression evoked by TGFβ in TME of GBM. Clinical studies utilizing dominant negative TGFRII (dnTGFRII) showed that dnTGFRII-engineered tumor-specific T cells therapy overcame tumor immune evasion and induced responses in Hodgkin lymphoma (24). Recently, dnTGFRII-modified CAR-T has also been applied in prostate cancer treatment (25). These studies support our notion of applying TGFβ-trap CAR-T in GBM therapy.

Thus, in this study we have engineered TGFRII ECD as the TGFβ-trap and selected EGFRvIII as the target in GBM to optimize and develop TGFβ-resistant CAR-T for GBM therapy. This innovative CAR-T enhanced the eradication of glioma cells and prolonged survival of glioma-bearing mice. Remarkably, CAR-T cells infiltration was significantly increased in the group treated with TGFβ-trap CAR-T; microglia profiling indicated that GBM-infiltrated microglia were polarized from tumorigenic toward a pro-inflammatory phenotype.

## Results

### TGFβ Is Overexpressed in Mouse GBM

**Figure 1:**
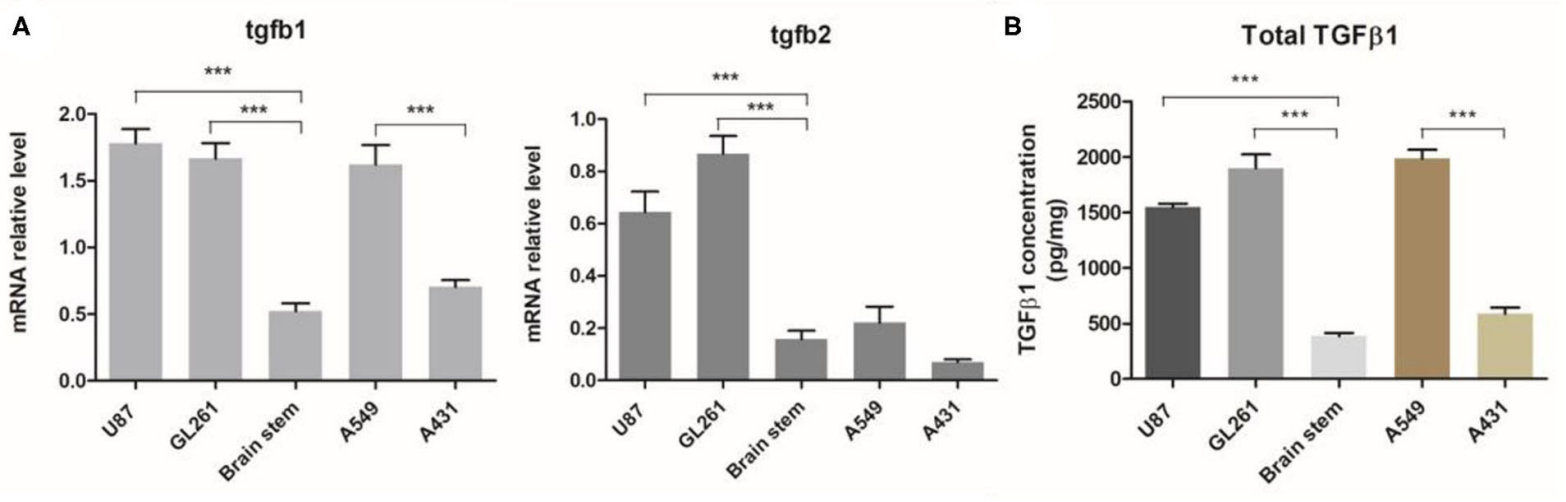
TGFβ expression in GBM and other aggressive tumors. Orthotopic GBM tumor and other xenograft aggressive tumors models were established. **(A)** The relative mRNA levels of TGFβ1 and TGFβ2 in tumor tissues or normal brain tissues (brain stem) were evaluated. **(B)** TGFβ1 protein production was measured by ELISA and normalized with total protein mass used for TGFβ1 detection. ***P < 0.001, one-way ANOVA followed by Tukey post-test.

Previous studies have reported that there was elevated expression of TGFβ1 and TGFβ2 in both blood serum and tumor tissues of patients with malignant glioma (14, 26, 27). To validate that our tumor models have a similar TGFβ-enriched TME, we intracranially established GBM tumor models with human glioma cell line U87 and mouse-derived GL261 cells in immunodeficient mice. The levels of TGFβ in GBM and other aggressive xenograft tumors including A549 lung cancer and A431 epidermoid carcinoma were measured. Compared to normal brain tissue, both TGFβ1 and TGFβ2 expression were dramatically increased in either U87 or GL261 tumors in mRNA (Figure 1A). In addition, total TGFβ1 protein level in GBM was three times more than that of normal brain tissues (Figure 1B), which is similar to what has been observed in GBM patients. TGFβ1 expression is significantly higher in A549 lung tumor, but not in A431 epidermoid carcinoma (Figure 1B), as reported before.

### TGFβ Represses T Cell Immune Response to Anti-CD3/CD28 Activation

**Figure 2:**
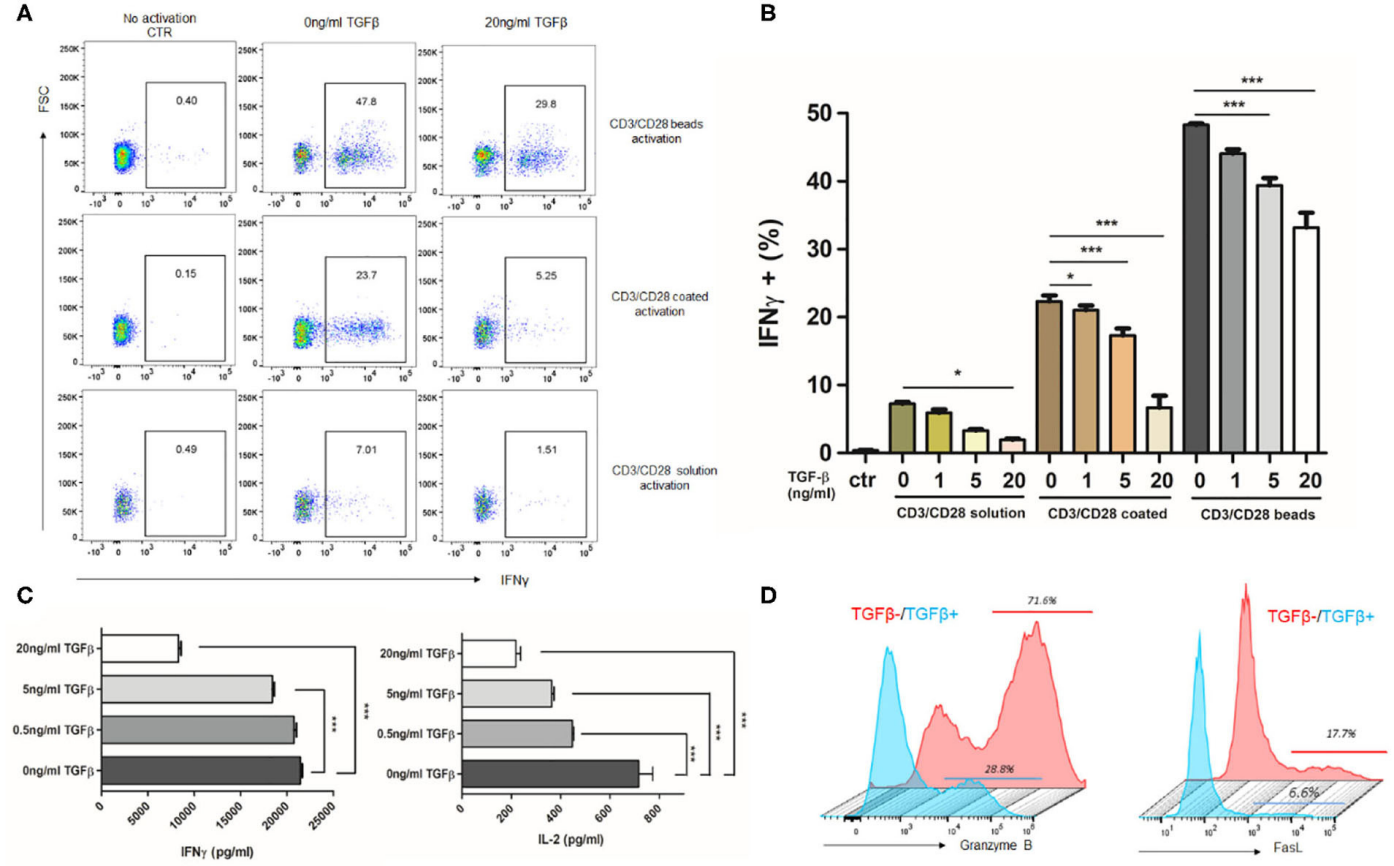
TGFβ represses T cell immune response to anti-CD3/CD28-induced activation. Different strength of stimuli induces distinct extent of T cell activation. TGFβ significantly represses T cell activation and cytokine release. Representative dot plot **(A)** showing the portion of IFNγ-expressing CD8^+^ T cells activated by different strength of stimuli in the presence of 20 ng/ml TGFβ. **(B)** Summary of inhibition effect of different concentration of TGFβ on T cell activation. **(C)** Quantification of IFNγ and IL-2 by ELISA after CD3/CD28 beads activation in the presence of TGFβ. **(D)** Representative histogram showing the expression of granzyme B and FasL in T cells with or without 20 ng/ml TGFβ response to CD3/CD28 beads. *P < 0.05, ***P < 0.001, one-way ANOVA followed by Tukey post-test.

TGFβ, as a vital immunosuppressive factor, dampens the immune response in GBM and promotes tumor evasion. To confirm the immunosuppressive potency of TGFβ, we assessed the T cells response to stimuli in the presence or absence of TGFβ. As different strengths of stimulation may change the profiles of the T cell response, we activated T cells with different stimuli. The strong and medium stimuli (anti-CD3/CD28 beads and anti-CD3/CD28 coated) resulted in increased IFN-γ, IL-2, granzyme B, and FasL expression. However, the weak stimuli (anti-CD3/CD28 solution) only caused a small amount of IFN-γ expression (Figures 2A,B). In the presence of TGFβ, the amount of IFN-γ-expressing CD8^+^ T cells dramatically decreased in a dose dependent fashion, regardless of stimuli type (Figures 2A,B). This was also reflected in levels of secreted IFN-γ by ELISA (Figure 2C). In addition, we observed that with strong stimulation, TGFβ inhibited IFN-γ, and IL-2 secretion (Figure 2C) of PBMC, and decreased the proportion of CD8^+^ T cells that express granzyme B and FasL (Figure 2D). These findings confirm that TGFβ is a potent immune suppression cytokine that inhibits T cell immune responses.

### Design and Functional Characterization of TGFβ-Trapped CAR T Cells

**Figure 3:**
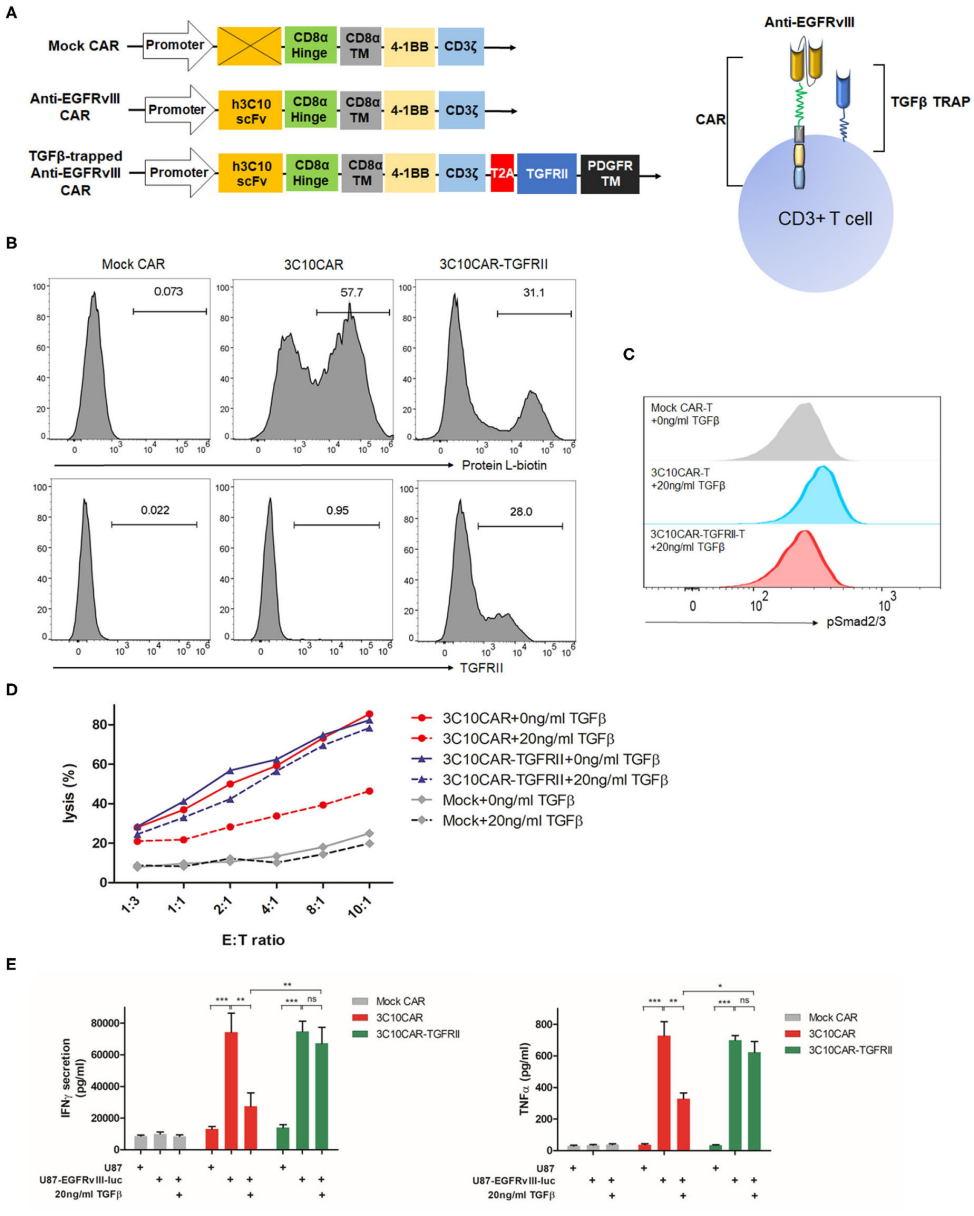
Design and functional characterization of TGFβ-trapped EGFRvIII-specific CAR T cells. **(A)** Left, illustration of gene structure of Mock CAR, anti-EGFRvIII CAR and TGFβ-trapped anti-EGFRvIII CAR; right, diagram of T cells armed with TGFβ-trapped anti-EGFRvIII CAR. **(B)** Representative histogram showing the transduction rate of PBMC with CARs-encoded lentivirus. CAR expression on T cells surface were detected by protein L-biotin. TGFRII expression were verified with recombinant TGFβ1. **(C)** Flow cytometry analysis of SMAD2/3 phosphorylation induced by TGFβ in anti-EGFRvIII CAR or TGFβ-trapped anti-EGFRvIII CAR T cells. **(D)** Cytotoxicity of mock CAR-Ts, anti-EGFRvIII (3C10) CAR-Ts, TGFβ-trapped anti-EGFRvIII CAR (3C10CAR-TGFRII) T cells against EGFRvIII^+^ target cells in the presence or absence of TGFβ. **(E)** TGFβ effect on IFNγ and TNF-α secretion from CAR-Ts in effector-target killing assay. CAR-Ts were cocultured with EGFRvIII^+^U87 or non-EGFRvIII U87 cells at the density of E:T ratio 1:1 for 24 h. *P < 0.05, **P < 0.01, ***P < 0.001 by two-way ANOVA followed by Bonferroni post-test.

Based on a second-generation CAR design, we engineered a Mock CAR construct lacking scFv, anti-EGFRvIII CAR, and TGFβ-trapped anti-EGFRvIII CAR, in each of which CD8α hinge, CD8α TM, 4-1BB costimulatory, and CD3z signaling domains were used, as shown in Figure 3A. The anti-EGFRvIII scFv was derived from one of the humanized 3C10 scFv, 2,173 construct, which has no cross-reactivity with wild-type EGFR (US2017/0008963A1). TGFRII ECD, as the TGFβ trap, was linked with PDGFR TM domain and anchored on cell membranes. In the TGFβ-trapped CAR construct, T2A peptide was embedded between CAR and TGFβ-trap components to ensure their co-expression. We transduced purified human PBMC with CAR lentivirus and evaluated the CAR and TGFRII EDC expression by flow cytometry using biotinylated recombinant EGFRvIII and TGFβ1. As shown in Figure 3B, we obtained a 30–60% transduction rate. CD4/CD8^+^ transduced T cells were further sorted for functional characterization. To determine the TGFβ-trap (TGFRII ECD) function of blocking TGFβ signaling, transduced CAR-T cells were respectively incubated with 0 ng/ml TGFβ1 or 20 ng/ml TGFβ1 for 30 min and Smad2/3 phosphorylation was detected by flow cytometry. In the presence of 20 ng/ml TGFβ1, phosphorylated Smad2/3 in 3C10CAR T cells has an incremental shift, but not in TGFβ-trapped 3C10CAR-TGFRII T cells (Figure 3C), indicating that TGFRII ECD on CAR-T cells can prevent TGFβ induced signaling transduction.

### TGFβ-Trapped CAR-T Retains Inherited Cytolytic Activity in TGFβ Context and Eliminates Target Cells *in vitro*

To characterize the cytolytic activity of TGFβ-trapped anti-EGFRvIII CAR T cells against GBM, we created an EGFRvIII-overexpressed cell line from human glioma U87 cells and designated U87-EGFRvIII-luc as the target cells, which constitutively expresses high levels of human EGFRvIII and luciferase (Supplemental Figure 1). Using this cell line, we conducted an LDH-release assay to evaluate cytotoxicity of CAR-T by co-culturing CAR-T cells with U87 or U87-EGFRvIII-luc target cells at varying effector-target (E:T) ratios. As shown in Figure 3D, compared to Mock CAR-T cells, 3C10CAR demonstrated potent killing efficiency. However, in the presence of 20 ng/ml TGFβ1, this cytotoxicity was dampened significantly. For TGFβ-trapped 3C10CAR-TGFRII T cells, 20 ng/ml TGFβ1 had only minimal inhibition of their cytolytic activity. To exclude non-specific killing, we also performed a cytolytic experiment with 3C10CAR-Ts or 3C10CAR-TGFRII Ts targeting non-EGFRvIII U87 cells. As shown in Supplemental Figure 2, both 3C10CAR and 3C10CAR-TGFRII CAR had minimum cytolytic activity on non-EGFRvIII U87 cells.

Next, we defined the cytokine profiles using a killing assay. 2 × 10^6^ CAR-T cells were cocultured with U87-EGFRvIII-luc target cells at an E:T ratio 1:1 with or without TGFβ for 24 h. Both 3C10CAR and 3C10CAR-TGFRII T cells responded to U87-EGFRvIII-luc target cells, leading to a substantial amount of IFNγ, IL-2, CCL4, TNF-α, and IL13 release. IL-4 and IL-5 were secreted at very low levels in either 3C10CAR or 3C10CAR-TGFRII T cells (Supplemental Figure 3). Comparative analysis revealed that IFNγ and TNF-α expression were significantly inhibited by TGFβ1 in 3C10CAR-T cells coculture, but not observed in the 3C10CAR-TGFRII T cells group (Figure 3E), which was in coordinate with the cytotoxicity disparity between 3C10CAR-T and 3C10CAR-TGFRII T cells. For other cytokines, such as IL-2, IL-13, and CCL4 (Supplemental Figure 3), 3C10CAR-T cells and 3C10CAR-TGFRII T cells shared similar profiles responding to U87-EGFRvIII-luc cells plus TGFβ.

In the long term of co-cultures with target EGFRvIII-U87 cells, both 3C10CAR-Ts and 3C10CAR-TGFRvIII Ts proliferated (Supplemental Figure 4). When cocultured with U87 cells proliferation was not observed, indicating that proliferation depended on EGFRvIII specific binding, but not TGFRII binding.

### Tumor Growth Is More Effectively Controlled by TGFβ-Trapped CAR-T in Orthotopic GBM Models

**Figure 4:**
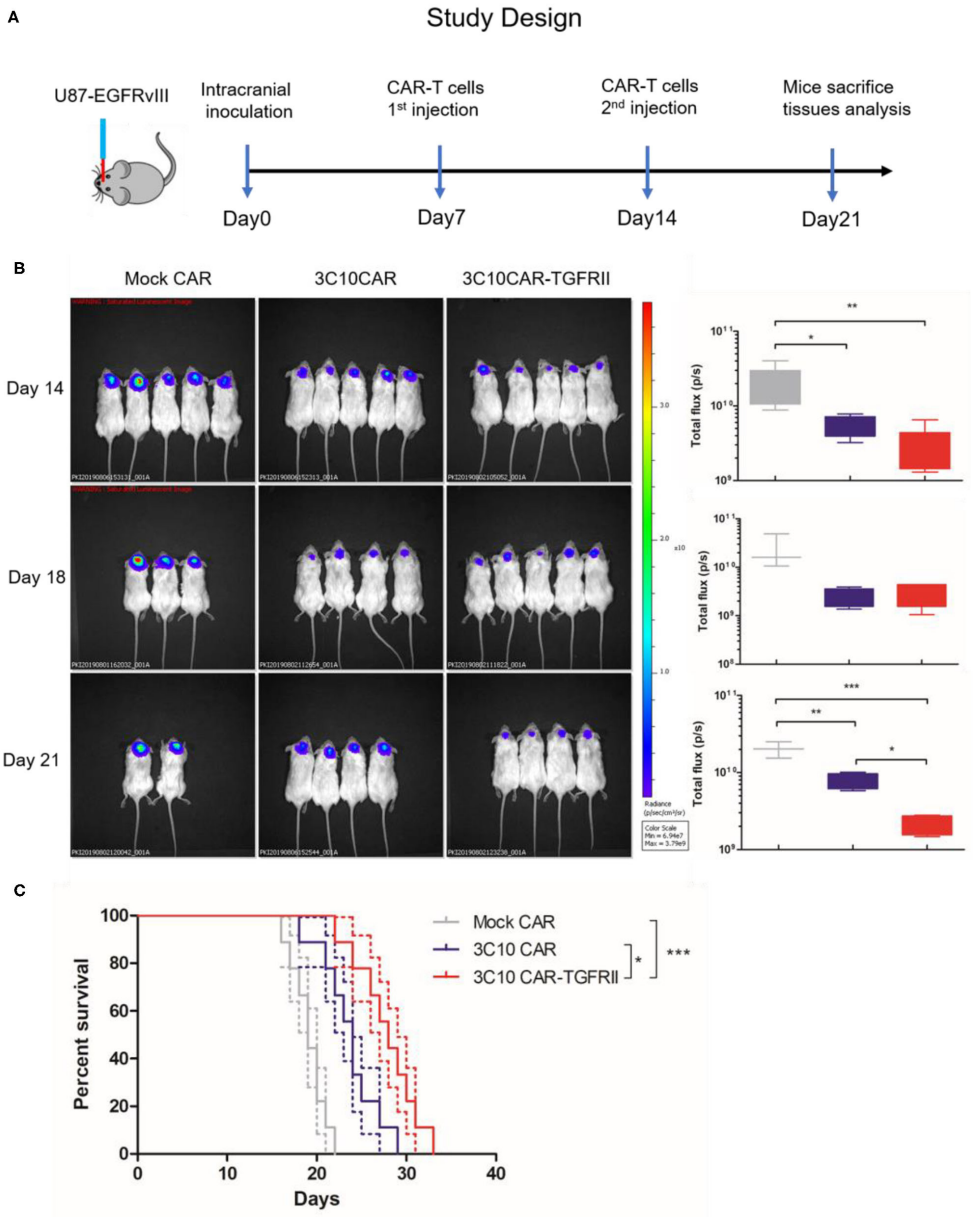
TGFβ-trapped anti-EGFRvIIII CAR-T more effectively controls tumor growth in orthotopic GBM models. **(A)** *in vivo* evaluation protocol for anti-tumor efficacy of CAR-Ts for GBM. NSG mice were inoculated intracranially with U87-EGFRvIII-luc cells on day 0. Mice were injected intravenously with CAR-Ts on day 7 and 14. **(B)** *In vivo* imaging illustrating the growth of U87-EGFRvIII-luc in CAR-Ts-treated mice. Average tumor flux was quantified in each group. **(C)** Kaplan-Meier survival curves of U87-EGFRvIII-luc-bearing mice treated with different CAR-Ts. *P < 0.05, **P < 0.01, ***P < 0.001 by Mantel-Cox test.

Next, we investigated the *in vivo* antitumor activity of 3C10CAR-T and 3C10CAR-TGFRII T cells in NSG mice bearing TGFβ-enriched human glioma tumors as described in Figure 4A. By injecting U87-EGFRvIII-luc cells intracranially, we established a GBM tumor model (day 0) which had been previously demonstrated to process comparable TGFβ expression. One week later, mock CAR, 3C10CAR, and 3C10CAR-TGFRII T cells were injected intravenously into mice. Bioluminescence imaging (Figure 4B) demonstrated that tumors had a significant reduction in both 3C10CAR and 3C10CAR-TGFRII groups on day 14. 3C10CAR-TGFRII demonstrated a slight but not significant benefit relative to 3C10CAR group. On day 14, mice were again injected with another shot of CAR-T cells. Seven days after the second shot of CAR-T (21 days after tumor implantation), nearly half the mice in the mock CAR-T group died (Figure 3C). Tumors in the 3C10CAR-TGFRII group remained quite small; while the 3C10CAR group showed slow tumor progression, suggesting that 3C10CAR-TGFRII Ts controlled tumor growth more efficiently and functioned better in TGFβ-enriched glioma. At 21 days post-tumor implantation, mice were sacrificed and brain tissues containing tumors were harvested for further analysis. In parallel experiments, we observed that 3C10CAR-Ts consistently prolonged the survival of mice as previously shown, whereas TGFβ-trapped 3C10CAR-TGFRII Ts performed significantly better than 3C10CAR and improved the anti-tumor benefit (Figure 4C). This implies that the structure of TGFβ-trap armed on 3C10CAR-T cells is helpful to enhance the anti-tumor activity of CAR-Ts in the context of TGFβ-enriched TME.

**Figure 5:**
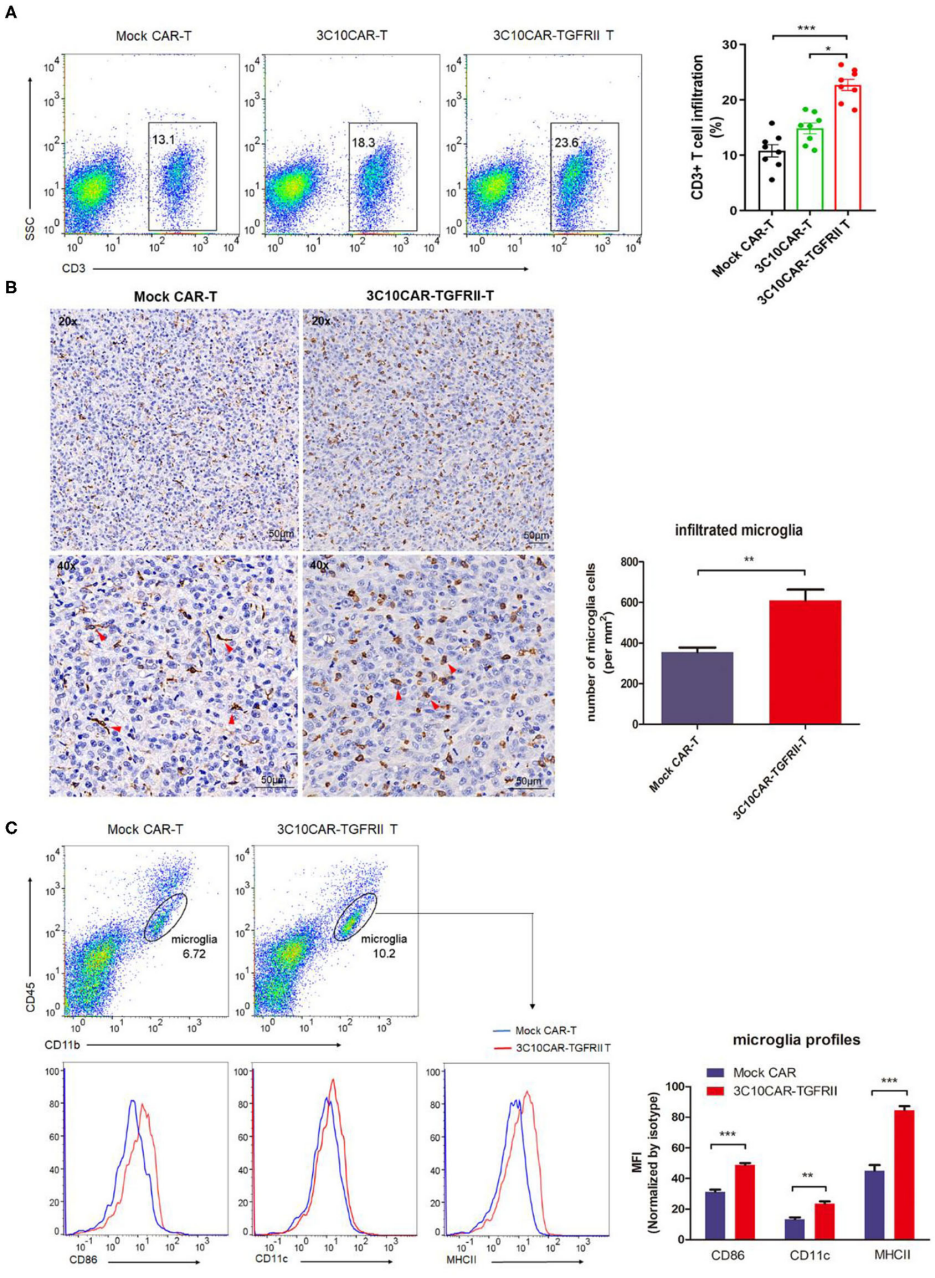
TGFβ-trapped CAR-T treatment increased CAR-Ts infiltration and convert infiltrated-microglia to pro-inflammatory phenotype. Glioma tissues were harvested and dissociated into single cell suspension. **(A)** Representative dot plots showing the proportion of infiltrated hCD3^+^ CAR-T cells in cells suspension (left); quantification of CD3^+^T cell infiltration in different treatment groups (right). **(B)** Immunohistochemistry of IbaI^+^ microglia in glioma tissue showing the number of infiltrated microglia increased in 3C10CAR-TGFRII group and morphology change (red arrow). **(C)** Flow cytometry of CD11b^+^ CD45^low^ microglia cells showing the expression of pro-inflammatory markers, CD86, CD11c, and MHCII in Mock CAR or 3C10CAR-TGFRII groups. Pro-inflammatory markers expression was quantified by MFI and normalized with isotype control. *P < 0.05, **P < 0.01, ***P < 0.001, **(A)** Is by one-way ANOVA followed by Tukey post-test. **(B,C)** Are by Two-tailed *t*-test with Welch's correction.

CAR-T cells infiltration into tumor sites is the critical indicator of prognosis of CAR-T therapy. To assess CAR-T infiltration, harvested glioma tissues were dissociated into single cell suspension for flow cytometry analysis. We observed that glioma tissue from the 3C10CAR-TGFRII group gave significantly higher number of T cells infiltration into glioma sites than that in the 3C10CAR group (Figure 5A), correlating with improved anti-tumor activity. Moreover, immunohistochemistry staining of Iba1^+^ microglia in GBM showed that the number of infiltrated microglia in tumors treated with 3C10CAR-TGFRII was significantly increased compared with that in the Mock CAR group (Figure 5C). In contrast to the ramified appearance of that in the mock CAR group, infiltrated microglia in the 3C10CAR-TGFRII group had morphology change and showed soma enlargement and process retraction (Figure 5B). Analysis of the M1/M2 profiles demonstrated these microglia showed elevated M1 markers (Figure 5C), including MHCII, CD11c, and CD86, indicating that the microglia polarized toward to M1, which is beneficial for an anti-tumor effect.

## Discussion

Although innovative therapeutics is emerging, GBM treatment remains difficult. A major obstacle for developing effective immunotherapy, including CAR-T therapy for GBM, is the extremely complex tumor microenvironment that compromises immune response by secretion of immunosuppressive cytokines including TGFβ (9, 28–30). Once autologous T cells or adoptive transferred CAR-T cells approach the tumor site, the local TME can affect the efficacy of these cells (31, 32). To overcome these limitations, here we modified the EGFRvIII-specific CAR with a TGFβ-trap targeting TME. In this manuscript, we demonstrated our TGFβ-trap EGFRvIII-specific CAR-T diminished the immunosuppressive effect *in vitro* and enhanced the anti-tumor activity *in vivo*.

Immunotherapy interventions for GBM include checkpoint inhibitors, viral therapy, vaccination with dendritic cells and adoptive cell transfer, among which CAR-T therapy has been demonstrated as promising in directing anti-tumor immune responses. CAR-Ts targeting IL-13Rα, HER-2, and EGFRvIII have been tested in clinical trials for GBM therapy. In the trials of IL-13Rα and HER-2-specifc CAR-Ts, sustained tumor regression or disease stabilization were observed in a few of patients (7, 33). Restricted expression pattern of the targeted tumor-associated antigens (TAA) is pivotal to the safety and successful application of CAR-Ts in GBM. In this regard, EGFRvIII is a favorable target antigen for GBM due to its restricted expression on tumor cells (34, 35). In a first-in-human study, ten patients intravenously infused with autologous EGFRvIII-specific CAR-Ts demonstrated its safety with no dose-limiting toxicity (8). Although CAR-Ts are also detectable in GBM regions, no measurable antitumor activity is documented.

These results highlight the critical issue of the efficacy of CAR-T cells targeting GBM. The immunosuppressive milieu of the GBM tumor microenvironment is proved to be the main hurdle for the anti-tumor function of CAR-Ts. Abundant evidence demonstrated that TGFβ was overexpressed in a GBM context and functioned as a determinant immunosuppressive factor. Therefore, creation of CAR-T cells that can modify the TME is a feasible strategy to improve CAR-T efficacy for GBM. We modified EGFRvIII-specific CAR with TGFβ-trap to target TGFβ in TME to accomplish this goal.

Our data confirms that TGFβ1 and TGFβ2 are all highly expressed in U87 or GL261-bearing orthotopic GBM models, which in turn indicates that these models are valid for mimicking the TGFβ-enriched context of GBM. The immunosuppressive function of TGFβ is validated by an *in vitro* assay. We observed that TGFβ intensively inhibits T cells activation and cytokine release.

We tested the cytotoxicity of our TGFβ-trapped EGFRvIII-specific CAR-T (3C10CAR-TGFRII Ts) in a TGFβ context *in vitro*. Data showed TGFβ-trapped CAR-Ts retained inherited cytolytic activity and had enhanced secondary cytokine release despite the presence of TGFβ relative to 3C10CAR Ts. Cytokine profiling indicated that TGFβ-trapped CAR-Ts polarized toward a Th1 phenotype with IFNγ and IL-2 expression. We also noticed that CCL4 and TNF-α expression increased when TGFβ-trapped CAR-Ts targeting U87-EGFRvIII-luc cells are compared to Mock CAR-Ts. Notably, studies reported that TNF-α generated by activated T cells mediated monocyte activation (36). Another study demonstrated that TNF-α increased EGFR expression in tumor cells (37). Thus, elevated TNF-α implies that TGFβ-trapped CAR-Ts treatment may contribute to enhancing innate immunity and preventing target antigen loss. Orthotopic inoculation of U87-EGFRvIII-luc cells mimics the GBM tumor microenvironment that enriches in TGFβ. Exploiting this model, the anti-tumor effect of TGFβ-trapped EGFRvIII-specific CAR-T was evaluated. *In vivo* data clearly demonstrated that the TGFβ-trap architecture enhanced anti-tumor capability of EGFRvIII-specific CAR-Ts in a TGFβ context and prolonged survival of GBM-bearing mice.

Infiltrated microglia comprise a considerable portion of tumor bulk mixture and are well-known to shape the complex microenvironment of GBM (38, 39). These tumor-associated microglia (TAM) involved in CSF-1, STI1, and especially TGFβ signaling, are reported to suppress immune response and promote glioma growth (40–42). Microglia-derived TGFβ enhances glioma invasion *in vitro* and *in vivo* (42). However, the characteristic of considerable diversity and plasticity of microglia indicates that microglia function in distinct roles. It is reported that pro-inflammatory microglia or M1 microglia demonstrate anti-tumorigenic potential (43). Re-polarization of pro-tumorigenic, anti-inflammatory M2 TAM to anti-tumorigenic M1 TAM can reinforce the anti-tumor immunity (44). In our study, we found that TGFβ-trapped CAR-Ts treatment increased microglia infiltration, but the M1/M2 balance of microglia seemed to shift to a pro-inflammatory M1 phenotype. IFNγ and TNF-α were produced when TGFβ-trapped CAR-Ts recognized target glioma cells. Given the fact that IFNγ and TNF-α typically polarize microglia toward pro-inflammatory type (45), we speculate that TGFβ-trapped CAR-Ts treatment may disturb the microenvironment of GBM and contribute to converting pro-tumorigenic microglia into a pro-inflammatory phenotype.

## Materials and Methods

### Mouse Models

All animal procedures were approved by Fudan University Institutional Animal Care and Use Committee. Male NOD-Prkdc^scid^Il2rg^em1^B2m^em1/Smoc^ mice (NSG) were purchased from Shanghai Model Organisms (Shanghai, China) and housed at Fudan University. To establish the orthotopic glioblastoma model, 3 × 10^5^ U87-EGFRvIII-luc cells or U87 cells in 5 μl PBS were injected intracranially into 8-weeks old NSG mice. Mouse was positioned in a stereotaxic apparatus and implanted with tumor cells at 2 mm lateral, 1 mm anterior to the bregma, and 3 mm ventral from the dura into the brain. On days 7 and 14, after surgery, mice were injected with 5 × 10^6^ MOCK-T or CAR-T in 100 μl PBS via tail vein for total 2 times. Bioluminescence imaging (BLI) was performed on IVIS Lumina 200 imaging station via intraperitoneally injection of luciferin to quantify brain tumor volumes on days 14, 18, and 21. Total pixels in each mouse was further analyzed. For subcutaneous models, 3 × 10^6^ A549 or A431 cells in 100 ul PBS were subcutaneously injected into NSG mice. Tumor size was measured by calipers in three dimensions, L × W × H, for the duration of the experiment. Tumor growth was monitored until the tumor volume reached 400 mm^3^. Mice were sacrificed and tumor tissues were collected for further analysis.

### Cell Lines and PBMC

HEK293FT cells and human lung carcinoma cell line A549 were provided by Dr. Fang Jianmin's lab from Tongji University. Human malignant glioma cell line U87, mouse malignant glioma cell line GL261 and human epidermoid carcinoma cell line A431 were purchased from the Type Culture Collection of the Chinese Academy of Sciences. U87-EGFRvIII-luc was created by lentivirus transduction of U87 cells with EGFRvIII and luciferase-GFP encoding lentivirus under control of CMV promoter. EGFRvIII-luciferase double positive single cell was sorted into 96-well plate and expanded to clones. Human peripheral blood mononuclear cells (PBMC) were isolated from the peripheral blood of healthy donors via Ficoll gradient. Isolated PBMC were cryopreserved for use. HEK293FT, U87, and U87-EGFRvIII-luc cell lines were cultured in DMEM (Thermo Scientific) supplemented with 10% fetal bovine serum (FBS). GL261 cells were maintained in DMEM/F12 with 10% FBS. PBMC were cultured in KBM581 serum-free medium (Corning).

### Vector Constructs

Humanized 3C10 scFv sequence from Dr. Jennifer Brogdon's patent application (US 20170008963 A1) were utilized as anti-EGFRvIII scFv. The TGFβ trap construct includes TGFRII extracellular domain from 1 to 159 residue anchored on cell membrane with PDGFRβ TM. Chimeric cDNA sequences containing humanized 3C10 scFv with CD8 signal peptide, CD8αTM-4-1BB-CD3z, and T2A-TGFRII-PDGFRβTM were respectively custom synthesized by Genscript. These modules were then cloned into the plvx-IRES-Zsgreen lentiviral vector via CloneExpress MultiS One step cloning kit (Vazyme). Human EGFRvIII sequence was acquired via overlap PCR from human EGFR cDNA by deletion of 6–276 amino acid. The spliced sequence was subcloned into plvx-IRES-puro vector. The lentiviral helper vectors pSPAX and pMD2.G were kindly provided by Dr. Fang Jianmin's lab from Tongji University.

### *In vitro* T Cell Transduction and Cultures

Lentiviral supernatants were harvested after 24 and 48 h transfection of HEK293FT cells and concentrated by centrifugation with Lenti-X concentration reagent (Takara). For *in vitro* functional analysis, CD4/CD8 T cells were purified from isolated PBMC by magnetic beads (Miltenyi) and transduced by CAR lentivirus. For animal experiments, PBMC were used for transduction. Isolated PBMC or T cells were stimulated with Human T-Activator CD3/CD28 Dynabeads (Invitrogen) for 24 h in serum-free KBM581 T cell medium supplemented with 50 U/ml rIL-2. The activated T cells were transduced by lentivirus in RetroNectin pre-coated plates. Transduced cells were replaced with fresh medium supplemented with 50 U/ml IL-2 every other day and expanded for 5 days. CAR-T cells were normalized to 30% or 50% CAR expression before functional evaluation.

### Enzyme-Linked Immunosorbent Assay

Orthotopic glioblastoma tissue U87, GL261 and xenograft A431, A549 tumor tissues were cut into small pieces and homogenized by mechanical homogenizer and sonication in NP40 lysis buffer with proteinase inhibitor cocktail (Thermo Scientific). The supernatant was harvested by centrifugation at 4°C. To determine the total amount of TGFβ, the supernatant samples were pre-treated with acidification to convert from latent to mature form. Total TGFβ was quantified by ELISA kit from Biolegend. Total protein concentration was determined by Pierce BCA protein assay kit from Thermo Fisher. The amount of TGFβ was normalized by dividing total protein mass used for TGFβ detection.

To determine the inhibitory effect of TGFβ on T cell activation, thawed naïve T cells were activated in different stimulation strengths by CD3/CD28 Ab solution, CD3/CD28 Ab-coated plate or CD3/CD28 beads in the presence of various concentration of rhTGFβ1, 0, 0.5, 5, or 20 ng/ml, respectively. After 24 h incubation, cell medium was harvested and IFNγ expression was evaluated by ELISA kit (Biolegend).

For cytokine analysis in cytotoxicity, mock CAR T, 3C10 CAR T, 3C10 CAR-TGFRII T were cocultured with target-expressing cells, U87 or U87-EGFRvIII-luc at an E:T ratio of 1:1 for 24 h in a 96-well U-bottom plates. The co-cultured cells were at the density of 1 × 10^5^ effector cells and 1 × 10^5^ target cells per well. Cell supernatants were harvested and cytokines secretion were analyzed by ELISA kit.

### Flow Cytometry

For all T cells staining, a fixable live/dead violet label (Invitrogen) was utilized to determine the live/dead ratio. Cells were stained with live/dead violet in PBS for 15 min, followed by surface staining in FACS buffer. APC/Cy7-labeled recombinant protein L was used to detect the CAR expression. For detection of TGFRII-PDGFRβTM, cells were stained with biotinylated hTGFβ1 and APC-strepavidin. Intracellular staining with AlexaFluor 647-pSmad2/3 (BD Bioscience) evaluated the TGFβ1-pSmad2/3 signaling transduction in 3C10 CAR or TGFβ-trapped 3C10 CAR T cells. To profile T cell response to stimuli in the presence of TGFβ1, intracellular IFNγ, IL-2, Granzyme B expression was evaluated. T cells were incubated with BFA for an additional 6 h and intracellularly stained with APC-IFNγ, PE-IL-2, APC-Cy7-Granzyme B. The following antibodies (Biolegend) for defining T cell phenotype defining were used: anti-CD3-FITC, anti-CD3-APC, anti-CD4-PE, and anti-CD8-APC/Cy7. For U87-EGFRvIII-luc cells sorting, anti-EGFRvIII antibody (DH8.3, Novo) and anti-mouse-IgG-APC were used to define EGFRvIII^+^ cells. Fluorescence was assessed by a 3-laser Invitrogen Attune NxT flow cytometer, and data was analyzed with FlowJo software.

### Cytotoxicity Assay

We used CytoTox-ONE (Promega) homogeneous membrane integrity assay to evaluate cytotoxicity of CAR-T cells. The effectors and targets were mixed together at the indicated E:T ratios with 2 × 10^4^ target cells and cultured in 96-well plates for 18 h in a total volume of 100 μl T cell medium per well. Target cells alone or effectors alone were seeded at the same cell density to determine the background LDH release. Maximum lysis was evaluated by adding 0.01% Triton X-100 lysis buffer into the same density of target cells. After 18 h, substrate was added to the wells and cells were incubated for 10 min, followed by stopping with stop solution. Fluorescence intensity was measured using EnVision plate reader.

### Statistics

All statistical analysis was performed using GraphPad Prism 5. Data were presented as means ± SEM as stated in the figure legend. For comparisons of two groups, two-tailed unpaired *t*-tests were used. One-way ANOVA with Tukey *post-hoc* test was used for comparison of three or more groups in a single condition. Kaplan-Meier survival data were analyzed using a log rank (Mantel-Cox) test.

## Data Availability Statement

The original contributions presented in the study are included in the article/Supplementary Material, further inquiries can be directed to the corresponding author/s.

## Ethics Statement

The animal study was reviewed and approved by Fudan University Institutional Animal Care and Use Committee.

## Author Contributions

YL and TY funded this work and designed all the experiments. YL, HW, and GC mainly conducted the *in vitro* and *in vivo* experiments. XW and SZ provided the cell lines and constructed the plasmid. CW, AH, and YW helped to revise the manuscript. ZZ and CZ provided the stereotaxic Instruments. All authors contributed to the article and approved the submitted version.

## Conflict of Interest

The authors declare that the research was conducted in the absence of any commercial or financial relationships that could be construed as a potential conflict of interest.

## Abbreviations

GBM: glioblastoma
TGFβ: transforming growth factor β
ECD: ectodomain
TMB: tumor microenvironment
CAR: Chimeric antigen receptor
EGFRvIII: Epidermal growth factor receptor variant III
TGFRII: transforming growth factor β receptor type II.
