# Supplementary material for: Arming Anti-EGFRvIII CAR-T With TGFβ Trap Improves Antitumor Efficacy in Glioma Mouse Models
Source: Front Oncol. 2020 Aug 18;10:1117. doi: 10.3389/fonc.2020.01117 (PMC7461942; doi:10.3389/fonc.2020.01117)
Supplement: Supplementary file 1 [file Data_Sheet_1.PDF]

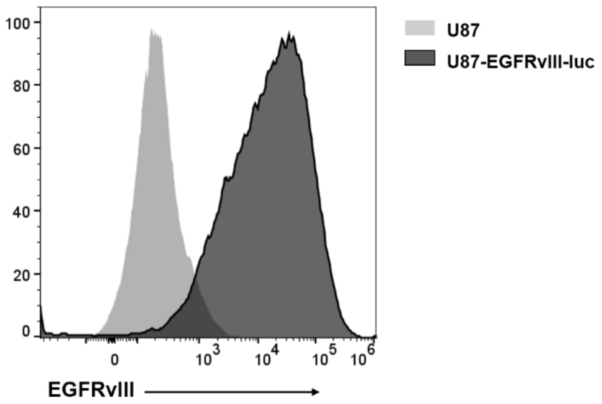

Supplemental Fig.1. Construction of EGFRvIII-overexpressed glioma cell line. Human glioma cell line U87 were transduced with EGFRvIII and luciferase encoding lentivirus. Histogram showing EGFRvIII overexpression in U87-EGFRvIII-luc cells.

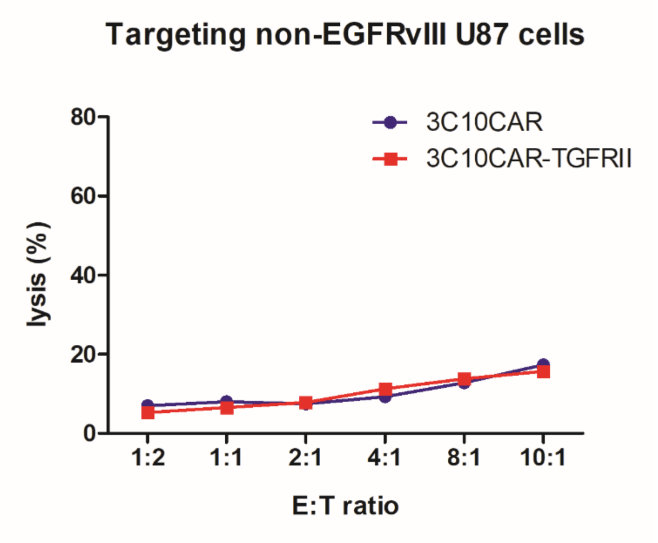

Supplemental Fig.2. Cytolytic activity of 3C10CAR-Ts or 3C10CAR-TGFRII-Ts targeting non-EGFRvIII U87 cells. Both 3C10CAR-Ts and 3C10CAR-TGFRII-Ts showed minimum cytolytic activity when coculturing with non-EGFRvIII U87 cells.

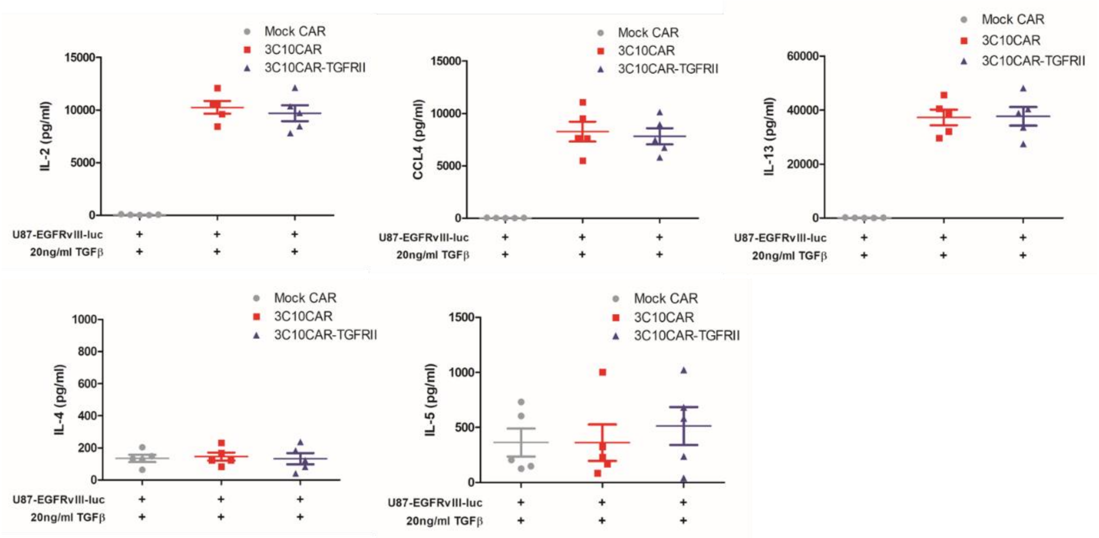

Supplemental Fig.3. Cytokine release pattern of CAR-T cells targeting U87-EGFRvIII-luc cells and TGFβ.

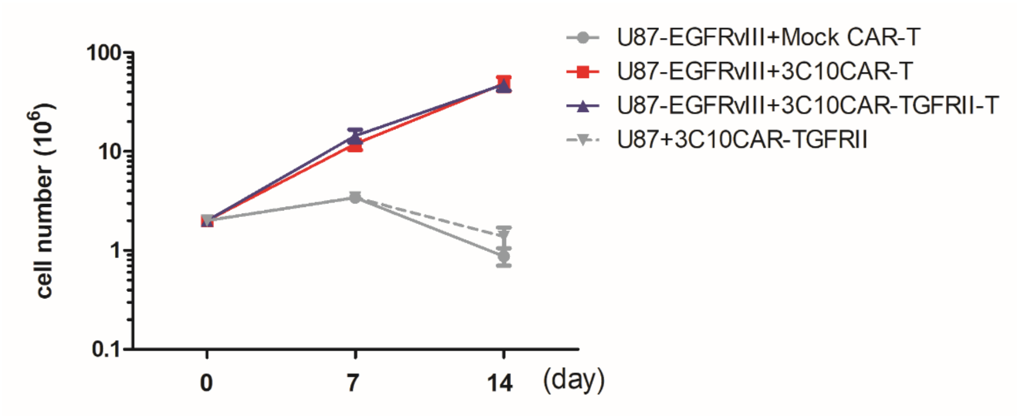

Supplemental Fig.4. CAR-T cells proliferation when cocultured with target cells.
